# Multiplex PCR based screening for micro/partial deletions in the AZF region of Y-chromosome in severe oligozoospermic and azoospermic infertile men in Iran

**Published:** 2015-09

**Authors:** Majid Motovali-Bashi, Zahra Rezaei, Fariba Dehghanian, Halimeh Rezaei

**Affiliations:** *Division of Genetics, Department of Biology, Faculty of Sciences, University of Isfahan, Isfahan, Iran.*

**Keywords:** *Y chromosome*, *Male infertility*, *Microdeletion*, *Azoospermia factor*

## Abstract

**Background:**

Infertility is a health problem which affects about 10-20% of married couples. Male factor infertility is involved approximately 50% of infertile couples. Most of male infertility is regarding to deletions in the male-specific region of the Y chromosome.

**Objective:**

In this study, the occurrence of deletions in the AZF region and association between infertility and paternal age were investigated in Iranian men population.

**Materials and Methods:**

To assess the occurrence of Y chromosomal microdeletions and partial deletions of the AZF region, 100 infertile men and 100 controls with normal spermatogenesis were analyzed. AZFa, AZFb, AZFc and partial deletions within the AZFc region were analyzed using multiplex PCR method. Finally, the association between paternal age and male infertility was evaluated.

**Results:**

No AZFa, AZFb or AZFc deletions were found in the control group. Seven infertile men had deletions as the following: one AZFb, five AZFc, and one AZFab. Partial deletions of AZFc (gr/gr) in 9 of the 100 infertile men (9/100, 9%) and 1 partial AZFc deletions (gr/gr) in the control group (1/100, 1%) were observed. In addition, five b2/b3 deletions in five azoospermic subjects (5/100, 5%) and 2 partial AZFc deletions (b2/b3) in the control group (2/100, 2%) were identified. Moreover, the risk of male infertility was influenced by the paternal age.

**Conclusion:**

The results of this study suggested that the frequency of Y chromosome AZF microdeletions increased in subjects with severe spermatogenic failure and gr/gr deletion associated with spermatogenic failure.

## Introduction

Infertility is estimated to affect 10–15% of couples, and roughly half of these cases are due to men’s problems. Spermatogenic failure is the most common form of male infertility, and the important role of the Y chromosome in the male infertility is increasingly recognized (1, 2). The male-specific region of Y chromosome (MSY) consists of long, Y-specific repeats called “amplicons”. Homologous recombination between amplicons has been shown to generate deletions, commonly resulting in spermatogenic failure. Three azoospermia factors (AZFa, AZFb, and AZFc) have been mapped on Yq11, and the AZFc region that completely comprised of amplicons is particularly susceptible to deletions. AZFc was defined as the most commonly known genetic factor that leads to azoospermia or oligozoospermia (3). Although it is difficult to establish precise genotype/phenotype correlations in patients with Y chromosomal microdeletions, deletions of AZFa or AZFb and deletions involving more than one region (AZFbc or AZFabc) have more severe effects on spermatogenesis than deletions of the AZFc region (4, 5). Partial deletions of the AZFc region (e.g. gr/gr or b1/b3) resulted from homologous recombination between amplicons within the AZFc region, and removing smaller numbers of genes compared to complete As ZFc deletions, have been described (6). The complete AZFc deletison (referred to as the b2/b4 deletion), which loses a segment of about 3.5Mb with eight testis-specific expressed gene families, has long been known to cause spermatogenic failure with few exceptions (7). After the construction of physical maps of the AZFc region, several types of AZFc partial deletions were identified, including the gr/gr subdeletion, the b2/b3 subdeletion (also referred to as the g1/g3 deletion or the u3-gr/gr deletion), and the b1/b3 subdeletion. The gr/gr subdeletion and the b2/b3 subdeletion are the most commonly detected. The gr/gr partial deletion, which is caused by nonallelic homologous recombination between g1/r1/r2 and g2/r3/r4 amplicons, leads to an excision of 1.6Mb DNA segment from the AZFc region (8). The results of studies on the roles of this deletion with spermatogenic failure have not been consistent. In Dutch, Spanish, Italian, and Australian populations, the gr/gr partial deletions have been reported as significant risk factors for spermatogenic failures (9-11). However, this partial deletion in other populations such predispositions has not been reported to date (12-18). The b2/b3 partial deletion, which removes a 1.8Mb DNA segment, is preceded by an inversion event. The gr/gr inversion is followed by a b2/b3 deletion, while the b2/b3 inversion is followed by a gr/gr deletion (19). The association between the b2/b3 partial deletion and male infertility has been recently reported in Chinese men, whereas no such a predisposition was detected in other populations (18-20). Other genetic events, such as the b1/b3 deletions, have a much lower frequency in the general population than the gr/gr and b2/b3 deletion, and their influence on spermatogenesis have not been identified. The exact effects of these partial AZFc deletions on spermatogenesis are still controversial. Some studies have shown a significant association between partial AZFc deletions and spermatogenic failures (21, 22), but others have not (12, 13). However, as the penetrance of these partial deletions is far lower than that of deletions involving the entire AZFc, it is to be expected that these partial deletions would not have severe effects on spermatogenesis. Data on the partial deletions of the AZFc region are still scant and indicate frequencies between 4% and 6% of men with spermatogenetic failure. In addition, some deletion patterns are more common in some populations, for example, in Eastern Siberian Yakuts and are compatible with normal spermatogenesis and fertility (12, 13, 21, 22). These studies suggest that geographical and ethnic differences might influence the frequencies of AZF deletions and of partial deletions of the AZFc region, as well as the deletion patterns and, possibly, the phenotypic expression. In addition, there is an overall belief that the fertility potential of older man is fairly well conserved. However, recent evidence supports the concept that increased paternal age is correlated with an increase in sperm chromosomal aneuploidy (23). The risk for father over 40 years old to have a child with an autosomal dominant mutation equals to the risk of Down syndrome for a child whose mother is 35-40 years old. Fathers older than 40 years had a 20% greater chance of having a baby born with a serious birth defect (24). In this study, the occurrence of Y chromosomal microdeletions and partial deletions of the AZFc region were assessed in an Iranian population. Also, association between paternal age and infertility was investigated.

## Materials and methods

**Study group**

This Case Control study consisted of an unselected group of 100 infertile men and 100 fertile men who attended to the Isfahan Fertility-Infertility Center and Royan Institute during the period of April to July 2013. Informed consent was obtained from each subject. All subjects and controls were of Iran ethnic group. The subjects were divided into two groups: 70 azoospermic (usually referred to “no sperm count”) men and 30 severely oligozoospermic men (sperm concentration <1 × 10^6^/mL). A questionnaire was given to each patient to collect demographic data, past medical and surgical history, including history of orchitis, testicular maldescent, testicular injuries, chemotherapy and radiotherapy, and habits concerning smoking and alcohol consumption. All patients were examined for the size, volume and consistency of the testis, varicocele and secondary sexual characteristics. Hormone profiles (serum follicle-stimulating hormone )FSH(, luteinizing hormone )LH( and testosterone) of all patients were collected. The control population consisted of 100 men with known fertility (at least one child, n= 76) and normospermic men (> 20× 10^6^/mL, n= 24). The semen analyses were done according to the World health organization (WHO) criteria (25).

**Yq microdeletion analyses by sequence tagged site (STS) polymerase-chain-reaction (PCR)-based strategy**

Blood samples were used for standard DNA extraction methods (26), and amplified in multiplex polymerase chain reaction (PCR). Each of these subjects was tested for four AZF loci: the STS primers were used for AZFa (sY84) AZFb (sY87) and AZFc (sY254, sY255). SRY14 was used as internal control and samples from normal fertile men without Y chromosome microdeletions and from healthy women were used as normal controls. In addition, blank served as negative control. A total of 50-100 ng of genomic DNA was used as template in 25 µL reaction mix, 1× amplification buffer, 1-1.5 mmol/L MgCl2, 1 mmol dNTPs, 10-20 pmol of each primer and Taq DNA polymerase (1 unit). After an initial denaturation step of 5 min, each PCR reaction was carried out at the annealing temperature specific for each primer pair, ended by an elongation step of 10 min and cooled to 4 ^º^C. On the basis of the size of the product obtained, the PCR products were separated on 1-2% agarose gels stained with ethidium bromide. In case of any failure in amplification of samples, two additional PCRs were performed to confirm the absence of the unamplified STSs.

**Screening for partial AZFc deletions**

Subjects who did not carry Y AZF microdeletions were screened for partial AZFc deletions. In this step, it was used multiplex-STS-PCR systems, including primers sY1291, sY1191, sY1201 and sY1161 and also single PCR reaction, including sY1206 primer to screen the partial deletions of the AZFc region. Because of similar length of PCR product of sY1191, sY1201, and sY1206 STSs, it was used a single PCR reaction for analyzing sY1206 STS. The primer sequences for STSs are available from Gen Bank under the accession numbers: sY1161, G66148; sY1191, G73809; sY1291, G72340; sY1206, G68331; sY1201. Each subject was screened with five STSs specific for the gr/gr region, sY1291 and sY1191. A gr/gr deletion was identified by the absence of amplification of marker sY1291 and presence of all other STS. The b2/b3 deletions were characterized by the absence of the STS sY1191 and the presence of all other STS. The b2/b3 deletions were characterized by the absence of the STS sY1191, sY1161 and sY 1291 and the presence all other STS. The b2/b4–entire DAZ deletion was identified by the positive result of sY1201 and the negative results of sY254, sY255, sY1191, sY1291 and sY1206 (27).

All negative PCR reactions were repeated for at least three times. The PCR reaction was performed in a reaction volume of 50 ml consisting of 50-100 ng genomic DNA, 10 pM of forward and reverse primers each, 10 mMdNTPs and 25 mM MgCl2 and the Taq Polymerase. Amplification started with an activation step of 15 min at 95 ^º^C, followed by 35 cycles of 30 s denaturation (94 ^º^C), 90 s annealing (57 ^º^C) and 60 s elongation (72 ^º^C), ended by an elongation step of 10 min and cooling to 4 ^º^C. PCR products were separated on 3% agarose gel and visualized by the ethidium bromide staining method.

**Association between paternal age and male infertility**

In order to analyze the effects of paternal age on male infertility, it was evaluated the frequency of microdeletions and gr/gr, b2/b4 and b2/b3 partial deletions in two groups, including patients with father younger than 40 years old and older than 40 years.

**Statistical analysis**

Partial deletions frequency in the patient and control groups were compared using the Chi square and odds ratio Tests on Statistical Package for Social sciences program (SPSS 22 Inc., Chicago, USA), and p values less than <0.05 considered to be statistically significant.

## Results

**Y chromosome microdeletions**

**Figure 1:**
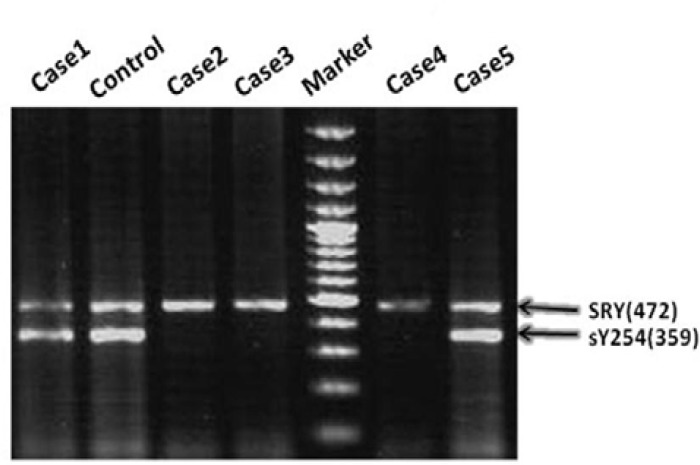
Electrophoresis results for detection of microdeletions in the AZFc region using STS-PCR. 472 bp fragments represent the control band, which it was amplified using SRY primers. Absence of amplified PCR products by sY254 primers results in the presence of microdeletions at AZFc region. Cases 2, 3 and 4 have got microdeletions, Cases 2 and 3 were azoospermia, and case 4 was oligozoospermia. Conditions of Electrophoresis were constant 55 Voltage including alternative current and 1.5% agarose gel. Marker: 100 bp DNA Marker

Infertile subjects included 70 azoospermic and 30 oligospermic men. The researchers did not screen the subjects with asthenozoospermia for microdeletions (Table I). Seven subjects were found to have microdeletions. One of these seven men (case 23) had severe oligozoospermia, and the rest had azoospermia. Microdeletions were not observed in control samples. All men with microdeletions had normal serum testosterone levels. Four patients had high serum FSH levels. One patient showed increased serum FSH and LH levels; the LH level was normal in the others. The deletions were present in 6 azoospermic men (6/70, 8.56%): four of them were idiopathic and two were cryptorchid (Table II). The overall frequency of microdeletions in infertile men was 7% (7/100). In azoospermic men, five deletions of the AZFc were detected: three of them had cryptorchidism. Two deletions involving the AZFc regions had Sertoli-cell-only syndrome (SCOS) in the testicular biopsy. The deleted STS loci and clinical characteristics of the subjects carrying microdeletions are shown in Table II. The further characterization of the microdeletions by extension analyses showed AZFb deletion in case 87. The microdeletion patterns were AZFc in five patients (cases 23,32,54,67 and 84) (Figure 1), and AZFbc in one patient (case 96) (Table II). No microdeletions were found in the AZFa region.

**Partial AZFc deletions:**

**Figure 2:**
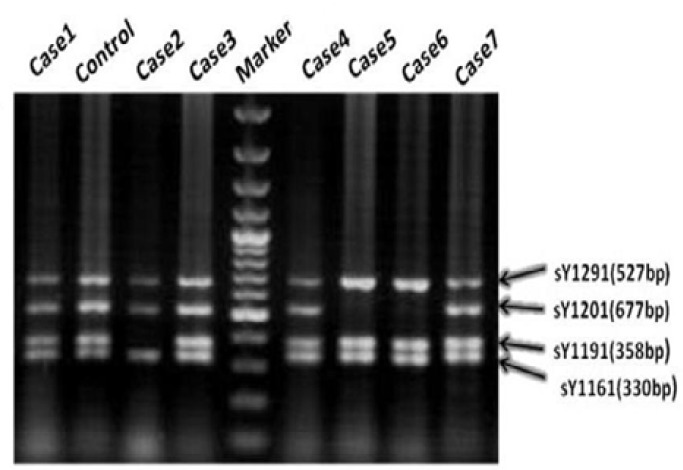
Detection of the gr/gr and b2/b3 partial deletions in the AZFc region using STS-PCR. Control: Normozoospermic man; Cases 2, 5, 6: The gr/gr deletions are defined by absence of sY1291 band and the presence of other STSs.

Two different patterns of partial deletions within the AZFc region were observed in five men in the patient group. Using AZFc specific STS markers it was identified nine cases of AZFc gr/gr deletions (absence of sY1291 and the presence of sY1191) among the infertile group (9/100, 9%) and one case (1/100, 1%) among the control groups (Figure 2) (Table III, IV). This difference was statistically significant. STS analyses identified b2/b3 deletions (absence of sY1191 and the presence of the marker sY1291) in five infertile men and two in the control group (Figure 2). In the infertile population, the gr/gr deletion was associated with azoospermia. The b2/b3 deletion was found in azoospermic men. Testicular biopsy reports were available for 4 of the 14 patients with partial deletions of the AZFc region. Two cases (cases 28 and 93) had SCOS and two patients (case 24 and 43) had seminiferous tubule atrophy. In the control population, it was found one normospermic man carrying the b2/b3 deletion (1/100, 1%) and two men of known fertility carrying the gr/gr deletion (2/100, 2%). All of them had normal ejaculate parameters (Table V).

**Effects of paternal age on male infertility**

The results in Table VI show that microdeletions in the patients with father over 40 years old were approximately 3.8 (P= 0.008) times more than the patients with father younger than 40 years old. In addition, the frequency of partial deletions, including gr/gr, b2/b4, and b2/b3 was 1.02 (P= 0.012) times more in the patients with father older than 40 years old (Table VI).

**Table I:** Frequency of Y chromosome microdeletion

**Phenotype**	**n**	**Deletion (%)**	**Region**
Azoospermia	70	6(6%)	AZFc,AZFb ,AZFc+AZFb
Oligosperima	30	1(1%)	AZFc
Total	100	7(7%)	

**Table II:** Clinical findings in seven infertile men with microdeletions in the azoospermia factor region

**Patient case No**	**Deletion pattern**	**Sperm concentration (×106)/mL) (nmol/L)**	**Testosterone**	**FSH (IU/L)**	**LH (IU/L)**	**Testicular histology**	**Clinic**	**STS deletion**
23	c	0.1	14.9	12.1	3.4	N/A	Cryptorchidism	sY254,sY255
32	c	0.0	12.6	23.5	2.7	SCOS	Cryptorchidism	sY254,sY255
54	c	0.0	24.2	4.3	5.9	Normal	Idiopathic	sY254,sY255
67	c	0.0	33.4	16.6	5.5	MA	Idiopathic	sY254,sY255
84	c	0.0	23.3	27.8	1.8	Normal	Idiopathic	sY254,sY255
87	b	0.0	29.7	4.4	3.9	N/A	Idiopathic	sY87
96	bc	0.0	21.3	87.0	23.3	SCOS	Cryptorchidism	sY254,sY255,sY87

FSH: Folliclestimulating hormone, LH: luteinizing hormone, MA: maturation arrest at the spermatid stage, N/A: not assessed, SCOS: Sertoli cell-only syndrome.

**Table III:** Partial azoospermia factor c deletions analyses in patients and controls

	**gr/gr deletion %**	**b2/b3 deletion %**	**Total partial AZFc deletions %**
Infertile	9/100(9)	5/100(5)	14/100(14)
Control	1/100(1)	2/100(2)	3/100(3)

**Table IV:** Clinical findings in six Iranian infertile men with partial deletions in the azoospermia factor c region

**Patient Testicular case No. (IU/L)**	**Deletion pattern histology**	**Sperm concentration (×106)/mL)**	**Testosterone**	**FSH (nmol/L)**	**LH (IU/L)**	**Testicular histology**
24	gr/gr	0.0	18.5	27.6	6.0	Atrophy
28	gr/gr	0.0	N/A	11.8	N/A	SCOS
51	gr/gr	0.0	9.9	63.9	34.0	N/A
78	gr/gr	4.2	23.3	4.3	2.1	N/A
93	b2/b3	0.0	8.8	25.0	5.4	SCOS
43	b2/b3	0.0	N/A	23.4	3.2	Atrophy

FSH: Folliclestimulating hormone, LH; luteinizing hormone, N/A; not assessed, SCOS; Sertoli cell-only syndrome

**Table V:** Semen parameters in men in the control group with partial deletions in the azoospermia factor c (AZFc) region

**Subject Morphology case No**	**Deletion pattern**	**Sperm (× 106/mL)**	**Concentration (a+b, %)**	**Motility (%)**
6	gr/gr	21.5	60.0	30.0
21	b2/b3	22.5	60.0	90.0
28	b2/b3	27.5	65.0	47.0

**Table VI:** Distribution of Y chromosome microdeletions and b2/b3, b2/b4, gr/gr partial deletions in patients with fathers older or younger than 40

		**Men with fathers younger than 40**	**Men with fathers older than 40**	**P value**	* **OR**	**95%CI**
	with Microdeletions (%)	2	11			
Microdeletions	without Microdeletions (%)	53	44	0.008	1.021	1.023-3.65
gr/gr, b2/b3,b2/b4 partial deletions	With Partial deletions (%)	5	11	0.012	3.686	1.502-6.15
	Without Partial deletions (%)	48	36			

* OR: 1.282-10.62

Case 2: The b2/b3 deletions are defined by absence of sY1191 and the presence of other STSs. Electrophoresis conditions were constant 40 Voltage, including alternative current and 2% agarose gel. Marker: 100 bp DNA Marker.

## Discussion

A number of genes on the Y chromosome and autosomes regulate spermatogenesis and Y chromosome deletions are emerging as a prevalent cause of male risk factor infertility (28). The frequency of Y chromosome deletions increases with the severity of spermatogenic defect (29). The frequency of deletions was reported to be in a range of 0.7%–34.5% in various studies (30). Furthermore, different frequencies and diverse patterns of Yq deletions, both microdeletions and partial deletions of the AZFc region, have been reported in different geographical regions and ethnic groups. In Europe, the overall AZF deletion frequency is approximately 8% of the men with non-obstructive azoospermia or severe oligozoospermia, affecting the AZFc region in most cases (31). Multi-region involvement (AZFbc or AZFabc) and deletions of AZFa were recorded at very low frequencies in the German population (32). The published data for Asia indicated certain variability in the deletion frequency depending on the selection criteria of the patients (33, 34). When the patients with azoospermia or severe oligozoospermia are considered together, the frequency of microdeletions varies from 5% in Eastern Uttar Pradesh in India (35), to 7.6% in Japan (36), 8.5% in Calcutta, India (37), 9% in China (38) and 10.6% in Taiwan, China (39). Interestingly, much higher frequencies of the AZFa deletions (17.2% in India) and the AZFbc deletions (51.7 % in India and 36.6 % in China) have been recorded, compared to those in Europe (37, 38). In the present study, it was reported the analyses of Y microdeletions in the Iranian population. We found that 7 of the 100 infertile Iranian subjects tested harbored microdeletion in the AZF region (7%). In three of the subjects (27), the AZF region deletion led to an azoospermic phenotype, can be suggest that deletions in these regions have an important role (3). The results were similar to the published data; the entire deletions of such the AZF region were associated with SCOS and spermatogenic arrest (28, 40). The deletions found in the present study concerned the AZFc and AZFb + AZFc regions. No deletions were found in the AZFa region. The frequency of AZF deletions in severe oligozoospermia was found to be lower than those in azoospermia. In the literature, the vast majority of deletions were found in azoospermic men with deletions frequency up to 7% (28). In men with sperm concentration less than 5 million, microdeletions were found sporadically, and in moderate oligospermia the deletions were rare (41). Several partial AZFc deletions have been described in earlier studies (9). These deletions resulted in the absence of several AZFc genes and in the case of the gr/gr deletion, it has been suggested to be an important genetic risk factor for spermatogenic failure (21).

Using a PCR approach, it was found the types of partial deletions, the gr/gr deletion and the b2/b3 deletion, in this study population. The gr/gr deletions were present in both controls (9%) and infertile (1%) men. This observation was statistically significant: the odd ratio test was performed and the P-value was 0.003, indicating difference in the occurrence of gr/gr deletions between the patients and the controls. This finding was similar to some recent data reporting a significant correlation between the presence of gr/gr deletion and spermatogenic failure in Spanish men (22). Hucklenbroich *et al*. (13) and Machev *et al*. (12) found no significant differences between the control group and the patient group. The b2/b3 deletion was detected in five (5%) patients and two (2%) control men. This observation was not statistically significant: the odd- ratio -tests was performed and the P-value was 0.054. The data were supported by several studies that have reported b2/b3 deletion in normospermic subjects. Although a strong correlation existed between classical AZFc deletions and spermatogenic failure, there was no evidence of a correlation between phenotype and genotype in the case of the gr/gr and b2/b3 deletions in our population (19, 21). Y chromosome AZF microdeletions were found at a frequency of 7% in the study population and consisted of AZFc or AZFb + AZFc deletions. In the partial AZFc deletions, we found b2/b3 deletions in infertile men and control groups, which suggested that this deletion may not be sufficient for spermatogenic failure in Iranian men. The gr/gr was observed in azoospermic men and it was possible to have an effect on spermatogenesis. In the present study, b2/b4–entire DAZ deletion was detected in 3 of the 100 subjects. This result suggested that the b2/b4–entire DAZ deletion might be a major AZFc abnormality responsible for impaired spermatogenesis among Iranian men, which is in agreement with reports in other populations. Some studies showed that increased age in infertile men was associated with an increasing in sperm DNA fragmentation and poor chromatin packaging, as well as with a decline in semen volume, sperm morphology and motility (42, 43). According to this study, it is suggested that increasing paternal age might be enhance cell division, mitosis, meiosis, and double strand break (DSB). So, intra-and inter-chromosomal recombination is occurred and some repeats in gene region is removed. This subject leads to spermatogenesis genes deletions and could be causing men infertility.

## Conclusion

In conclusion, on the basis of the present results, AZFc microdeletions seemed to be less frequent than the other patterns in Iranian men. b1/b3 deletion in patients and control samples was not observed. sY1201 deletion was detected in both patients (5 %) and control (2 %) men.sY1201 deletion was not detected in any population.

## Note

***This article extracted from student’s thesis*** ***(Zahra Rezaei)***
